# Comparison of different laboratory tests in the evaluation of hemorrhagic risk of patients using rivaroxaban in the critical care setting: diagnostic accuracy study

**DOI:** 10.1186/s12959-017-0140-6

**Published:** 2017-08-15

**Authors:** Marjorie Paris Colombini, Priscilla Bento Matos Cruz Derogis, Valdir Fernandes de Aranda, João Carlos de Campos Guerra, Nelson Hamerschlak, Cristóvão Luis Pitangueiras Mangueira

**Affiliations:** 1 Department of Diagnostic and Preventive Medicine and Clinical Laboratory, Hospital Israelita Albert Einstein, São Paulo, Brazil; 2 Department of Hematology, Hospital Israelita Albert Einstein, São Paulo, Brazil

**Keywords:** Anticoagulants, Direct oral anticoagulants, Rivaroxaban, Clinical laboratory techniques, Prothrombin time, Russell’s viper venom time

## Abstract

**Background:**

Rivaroxaban is a direct oral anticoagulant designed to dispense with the necessity of laboratory monitoring. However, monitoring rivaroxaban levels is necessary in certain clinical conditions, especially in the critical care setting.

**Methods:**

This is a diagnostic accuracy study evaluating sensitivity and specificity of prothrombin time (PT), activated partial thromboplastin time (aPTT), and Dilute Russell viper venom time (dRVVT), to evaluate the hemorrhagic risk in patients taking rivaroxaban. The study used a convenience sample of 40 clinically stable patients using rivaroxaban to treat deep vein thrombosis or atrial fibrillation admitted in a private hospital in Brazil, compared to a group of 60 healthy controls. The samples from patients were collected two hours after the use of the medication (peak) and two hours before the next dose (trough).

**Results:**

The correlation with the plasmatic concentration measured by anti-FXa assay was higher for PT and dRVVTS. The PT and aPTT tests presented higher specificity, while dRVVT was 100% sensible.

**Conclusions:**

There was a strong correlation between the tests and the plasma concentration of the drug. Additionally, our results demonstrated the potential use of dRVVT as a screening test in the emergency room and the need of a second test to improve specificity.

## Background

The traditional anticoagulant drugs that exist for the prevention and treatment of venous thromboembolism (VTE), prevention of cerebral vascular accident in patients with atrial fibrillation (AF), and secondary prevention in patients with acute coronary syndrome (ACS) require constant laboratory monitoring, which is sometimes burdensome and inconvenient for the patient. Rivaroxaban is a direct oral anticoagulant (DOAC) that dispenses with this type of control. It is an antithrombotic drug, that acts directly inhibiting activated factor X, impeding the generation of thrombin, and consequently preventing the formation of clots. It also has the advantage that it can be administered in a single daily dose [1].

Rivaroxaban has high bioavailability after oral administration, with a maximum peak of action at around 1.5 to 2 h after use (peak action), a mean half-life of between 5 and 9 h in young patients and 12 to 13 h in those aged over 75 years. It is eliminated in two ways: two thirds are metabolized by the liver (via CYP3A4 and CYP2J2) without any circulating active metabolite identified, and one third is excreted unaltered in the urine [2, 3].

Although direct oral anticoagulants have been designed to dispense the necessity of laboratory monitoring, the literature has demonstrated that this monitoring is potentially useful in certain conditions [2, 4, 5].

So far, it is known that rivaroxaban can prolong the times in the conventional clotting tests used to evaluate the risk of hemorrhage, such as prothrombin time (PT), activated partial thromboplastin time (aPTT), and thrombin time (TT), but it has not yet been possible to establish a therapeutic window of clinical interest that can be linearly correlated with the plasma concentration of the drug for these tests. The only two specific tests available to date, and that present correlation with the plasma concentration of the drug, are anti-FXa assay (indirect method) [1, 4], and liquid chromatography/mass spectrometry (direct method).

More recently, some authors have considered PT [6, 7] and dilute Russell viper venom time (dRVVT) [8–11] as the most promising tests for this type of monitoring. dRVVT evaluates only the common laboratory coagulation pathway test (factors X, V, II and I) after activation of factor X by Russell viper venom, minimizing the possibility of interference of dysfunction of the other clotting factors. There are also two different types of reagents in terms of the concentration of phospholipid used: the screen test (lowest concentration of phospholipid, dRVVTS) and the confirm test (highest concentration, dRVVTC) to evidence the presence of lupus anticoagulant. Exner et al., suggested that dRVVT could possibly be used to detect and maybe determine the plasmatic concentration of many anticoagulants, including rivaroxaban [11]. Douxfils et al. state also that the “russell viper venom time test” allows a rapid estimation of the intensity of anticoagulation mediated by rivaroxaban, although the authors did not differentiate the drugs type [12].

Altman and Gonzalez published a study in which they proposed that Russell’s viper venom is the most sensitive for identifying patients at risk of hemorrhage or exhibiting low anticoagulant effect, but they emphasize the need for other studies, to establish the sensitivity of other methods and identify cut-off values [8].

### Objective

The primary objective of our work was to compare the sensitivity, specificity, positive predictive value (PPV) and negative predictive value (NPV) of PT, aPTT and dRVVT, in order to exclude qualitatively the plasma concentrations that are relevant in critical care. The secondary objective was to correlate PT, aPTT, dRVVT (screen and confirm) assays with plasmatic concentration of the drug measured by the anti-Xa methodology in the same patients.

## Methods

### Study design, setting and ethics

This is a diagnostic observational study, conducted at a private hospital in São Paulo, Brazil, with a convenience sample of stable patients using the drug rivaroxaban.

The Institutional Review Board approved the protocol in advance under CAAE number: 34661614.1.0000.0071. All the subjects signed an informed consent form to perform blood tests and to participate in the study.

### Subjects

The subjects of this study are all patients consecutively admitted in the period of September 22, 2014 to December 21, 2015, in daily use of rivaroxaban at daily doses of 10, 15 or 20 mg daily (single dose), due to diagnosed thrombosis of the lower limbs, or those at risk of embolism due to atrial fibrillation. Patients with creatinine clearance lower than 15 mL/min or using rivaroxaban twice-daily regiment were excluded.

Plasma of 60 healthy controls without any known defects in the blood coagulation was used to estimate the normal range for the PT, aPTT and dRVVT screen and confirm assays.

### Laboratorial analytical methods

The patients’ blood samples were collected on two separate occasions: two hours after the use of the medication (“peak” moment) and two hours before the next dose (“trough” moment). The samples were collected according to the norms recognized by the Clinical Laboratory of the hospital, in test tubes containing 3.2% sodium citrate anticoagulant (Sarstedt, Newton, NC). Conventional PT tests were performed with the reagent STA Neoplastine CI Plus 10 and aPTT with STA Cephascreen 4 and processed on a Stago STA-R Evolution coagulation analyzer (Stago, Asnières-sur-Seine, France).

The Russell viper venom test was processed with the reagent STA Staclot DRVV (Screen and Confirm; Stago, Asnières-sur-Seine, France), at different phospholipid concentrations; one low, designated dRVVTS, and the other with a high concentration, designated dRVVTC.

The evaluation of anti-FXa assay was performed with the reagent STA-Liquid Anti-FXa with specific calibrator for rivaroxaban (Stago, Asnières-sur-Seine, France).

### Definitions

In this study, the following definitions were used:

- ≤ 30 ng/mL as the plasma concentration cut-off defined as safe for invasive procedures [13, 14];
- ≤ 50 ng/mL as the plasma concentration indicating moderate risk cut-off defined by Lim et al. [15] and Levy et al. [16];
- ≤ 100 ng/mL as the concentration that may lead to thrombolysis in ischemic stroke [14, 17];
- sensitivity as the proportion of individuals with a higher plasma concentration than the threshold (30 ng/mL, 50 ng/mL or 100 ng/mL) and a test result above normality;
- specificity as the proportion of individuals with a plasma concentration lower than the threshold (30 ng/mL, 50 ng/mL or 100 ng/mL) and a test result within normality range;
- positive predictive value (PPV) as the proportion of individuals with a positive test result who actually present plasma concentrations higher than the threshold (30 ng/mL, 50 ng/mL or 100 ng/mL);
- negative predictive value (NPV) as the proportion of individuals with a negative test result who do really present plasma concentrations lower than the threshold (30 ng/mL, 50 ng/mL or 100 ng/mL);
- correlation grades: coefficients < 0.2 meaning very weak correlation; 0.2 to 0.39 as weak correlations; 0.40 to 0.59 as moderate; 0.60 to 0.79 strong correlation; > 0.80 very strong [18].

### Statistical analysis

The plasmatic concentrations were described as means, standard deviation and 95% confidence intervals (CI). Spearman’s correlations were calculated between the anti-FXa assay values (above the lower limit of quantitation, LOQ, 25 ng/mL) and the laboratory tests of interest. The results were illustrated using scatter plots. The analyses were performed with the software GraphPad Prism 5 (GraphPad Software, San Diego, CA), considering a level of significance of 0.05. The results from healthy subjects were analyzed using the EP Evaluator 11.1.0.26 (Data Innnovations, LLC) software program. EP Evaluator follows the recommendations of the CLSI C28-A (Clinical and Laboratory Standards Institute guideline) [19]. The normal ranges were expressed in the 90% CI. The diagnostic test evaluation was calculated using the free online MEDCALC easy-to-use statistical software. CI for sensitivity and specificity are “exact” Clopper-Pearson CI; and, for the predictive values, the standard logit CI given by Mercaldo et al. [20] were used.

## Results

### Study group

In the study period, 40 patients required the use of anticoagulation and were included and evaluated (Table 1).

**Table 1:** Patients’ baseline data

Gender	n (%)
Female	20 (50)
Male	20 (50)
Rivaroxaban dosage	n (%)
10 mg/day	12 (30.0)
15 mg/day	19 (47.5)
20 mg/day	9 (22.5)
Age	
Average (standard deviation)	69 (21)
Minimum and maximum age	27–95

### Plasmatic concentration

The plasmatic concentration calculated by anti-FXa assay were as expected in trough, mainly less than the LOQ (25 ng/mL), for all dosages. In peak time, the results were: 63.4 ± 61.0 ng/mL (mean ± standard deviation, SD, with 95% CI: 24.7–102.1) for 10 mg; 142.5 ± 93.2 ng/mL (CI: 97.6–187.4) for 15 mg, and 203.4 ± 85.6 ng/mL (CI: 137.6–269.2) for 20 mg.

### Correlation between different methods with plasmatic concentration

**Fig. 1:**
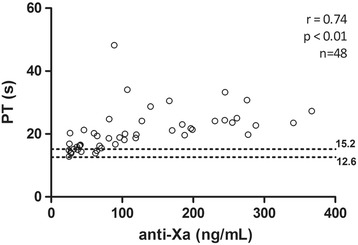
Correlation between anti factor X (anti-FXa) assay and prothrombin time (PT). Dotted lines indicate the normal range, determined from the control group

**Fig. 2:**
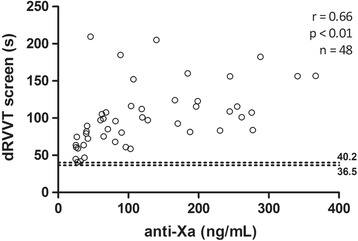
Correlation between anti factor X (anti-FXa) and dilute Russell viper venom time (dRVVT) screen assays. Dotted lines indicate the normal range, determined from the control group

**Fig. 3:**
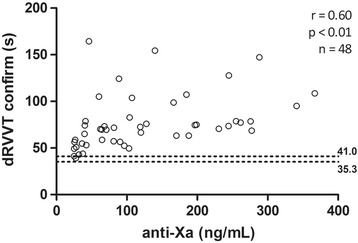
Relationship between anti factor X (anti-FXa) and dilute Russell viper venom time (dRVVT) confirm assays. Dotted lines indicate the normal range, determined from the control group

**Fig. 4:**
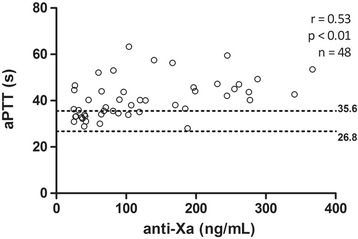
Correlation between anti factor X (anti-FXa) assay and activated partial thromboplastin time (aPTT). Dotted lines indicate the normal range, determined from the control group

All results of PT, dRVVT screen and confirm (Figs. 1, 2 and 3) presented a strong positive correlation with anti-FXa assay (*r* ≥ 0.60). The correlation of aPTT (Fig. 4) was only moderate (*r* = 0.53).

### Normal range

Table 2 shows the normal range calculated for TP, aPTT, dRVVs and dRVVc.

**Table 2:** Normal range calculated using samples from 60 health volunteers

Tests	Lower limit (90% confidence interval)	Upper limit (90% confidence interval)
PT (seconds)	12.6 (12.37–12.85)	15.2 (14.96–15.44)
aPTT (seconds)	26.8 (26.1–27.5)	35.6 (34.7–36.6)
dRVVT screen (seconds)	36.5 (36.2–36.9)	40.2 (39.9–40.6)
dRVVT confirm (seconds)	35.3 (34.8–35.8)	41.0 (40.4–41.5)

### Sensitivity, specificity, PPV and NPV

**Fig. 5:**
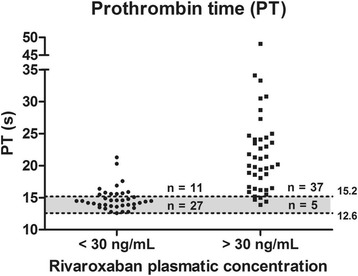
Prothrombin time (PT; seconds) distribution according to rivaroxaban plasmatic concentration. Normal range is represented by the gray band with a lower limit of 12.6 s (90% confidence interval, CI: 12.37–12.85) and upper limit of 15.2 s (90% CI: 14.96–15.44)

**Fig. 6:**
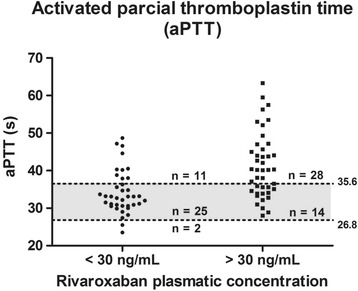
Activated partial thromboplastin time (aPTT), (seconds) distribution according to rivaroxaban plasmatic concentration. Normal range is represented by the gray band with a lower limit of 26.8 s (90% confidence interval, CI: 26.1–27.5) and a upper limit of 35.6 s (90% CI: 34.7–36.6)

**Fig. 7:**
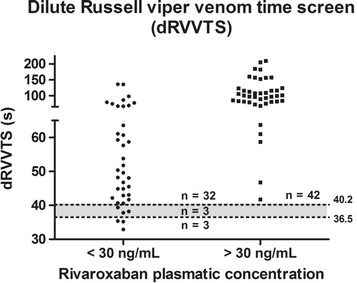
Dilute Russell viper venom time screen (dRVVTS) (seconds) distribution according to rivaroxaban plasmatic concentration. Normal range is represented by the gray band with a lower limit of 36.5 s (90% confidence interval, CI: 36.2–36.9) and upper limit of 40.2 s (90% CI: 39.9–40.6)

**Fig. 8:**
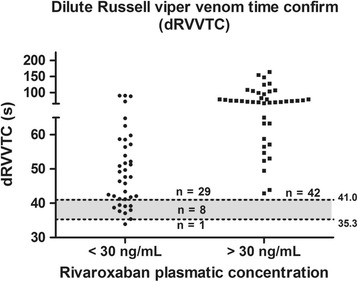
Dilute Russell viper venom time confirm (dRVVTC) (seconds) distribution according to rivaroxaban plasmatic concentration. Normal range is represented by the gray band with a lower limit of 35.3 s (90% confidence interval, CI: 34.8–35.8) and upper limit of 41.0 s (90% CI: 40.4–41.5)

Rivaroxaban concentration < 30 ng/mL was observed in 38/80 samples; < 50 ng/mL, in 46/80; and <100 ng/mL, in 57/80. Figs. 5, 6, 7, 8 and Tables 3, 4, 5 show the results dispersion and the performance of different tests according to different thresholds. It is possible to observe that, as the threshold increases, the TP and aPTT sensitivity also increases and specificity is reduced. dRVVs and dRVVc had the highest sensitivity regardless of the threshold adopted.

**Table 3:** Performance of four different tests for rivaroxaban monitoring (threshold: 30 ng/mL)

Test	Sensitivity (%, 95% CI)	Specificity (%, 95% CI)	PPV (%, 95% CI)	NPV (%, 95% CI)
PT	88 (74–96)	71 (54–85)	77 (67–85)	84 (70–93)
aPTT	67 (50–80)	71 (54–85)	72 (60–81)	66 (55–76)
dRVVs	100 (92–100)	16 (6–31)	57 (53–60)	100
dRVVc	100 (92–100)	24 (11–40)	59 (41–64)	100

*PPV* positive predictive value, *NPV* negative predictive value, *﻿CI*﻿ confidence interval

**Table 4:** Performance of four different tests for rivaroxaban monitoring (threshold: 50 ng/mL)

Test	Sensitivity (%, 95% CI)	Specificity (%, 95% CI)	PPV (%, 95% CI)	NPV (%, 95% CI)
PT	94 (80–99)	65 (50–79)	67 (57–75)	94 (79–98)
aPTT	76 (59–89)	72 (57–84)	67 (55–77)	80 (69–89)
dRVVs	100 (90–100)	13 (5–26)	46 (43–49)	100
dRVVc	100 (90–100)	20 (9–34)	48 (44–51)	100

*PPV* positive predictive value, *NPV* negative predictive value, *CI* confidence interval

**Table 5:** Performance of four different tests for rivaroxaban monitoring (threshold: 100 ng/mL)

Test	Sensitivity (%, 95% CI)	Specificity (%, 95% CI)	PPV (%, 95% CI)	NPV (%, 95% CI)
PT	100 (85–100)	56 (42–69)	48 (41–55)	100
aPTT	100 (85–100)	67 (53–79)	55 (46–64)	100
dRVVs	100 (85–100)	11 (4–22)	31 (29–33)	100
dRVVc	100 (85–100)	16 (8–29)	32 (30–35)	100

*PPV* positive predictive value, *NPV* negative predictive value, *CI* confidence interval

## Discussion

Among the direct oral anticoagulants (DOACs), rivaroxaban has been widely used in medical practice, particularly because it dispenses with the necessity of laboratory control in the majority of patients [4, 5, 8]. However, in conditions where a laboratory evaluation is desirable, it has been demonstrated by many authors [1] that the test of choice, due to its high specificity, is the plasmatic concentration obtained by the chromogenic anti-FXa assay with specific calibrator for rivaroxaban.

According to Baglin et al. [21], three methodologies can be additionally used for rivaroxaban monitoring: activated partial thromboplastin time (aPTT), prothrombin time (PT) and plasmatic concentration determination. Recent data pointed dRVVT as an additional tool [8, 9].

Levy et al. [10] cited that is very important to know when the last dose of the direct oral anticoagulant was taken by the patient in order to determine whether the levels are likely to increase or fall over time. There are some situations, however, in that obtaining this information is impossible, and this happens frequently in the emergency setting [14]. Besides, some patients might metabolize anticoagulant drugs differently. Therefore, it is important to know the performance of the evaluation techniques that are available in the laboratory, so that a correct approach can be used in the emergency room. Because of that, this study sought to evaluate the correlation and performance of different tests regarding the plasmatic concentration of rivaroxaban.

The plasmatic concentrations measured by anti-FXa assay were comparable to Mueck et al. [22]. Our results also show that PT was most closely correlated to plasmatic concentration measured by anti-FXa assay, followed by dRVVTs, dRVVTc and aPTT. The PT and aPTT correlation to plasmatic concentration was strongly discussed by Francart et al. [23], depending on reagent type. Douxfils et al. studied the response of two PT reagents and correlated PT data to the plasma concentration measured by HPLC-MS/MS. Correlation was found to be very strong (0.86) [9]. The differences between theirs and our results can be attributed to the characteristics of the patients evaluated, the reagents used and the methodology used in the plasmatic concentration measurement.

Gosselin et al. [10] correlated dRVVT to plasmatic concentration obtained by the direct method of liquid chromatography/mass spectrometry. They used the Siemens LA2 and Precision Biologics DRVVT reagents and presented a higher correlation coefficient (0.85 and 0.88, depending on the reagent). As for the PT and aPTT assays, the differences found can be attributed to the reagents, methods and even patients evaluated. In our study, if we isolate the results obtained with patients using 20 mg/day (*n* = 18), the correlation coefficient with anti-FXa assay would be 0.82.

Ebner et al. [14] recently described two thresholds of plasma concentration that are relevant in situations of emergency (30 and 100 ng/mL). Levy et al. [16] added the threshold of 50 ng/mL in cases of patients with severe bleeding. Lim et al., in 2016, described the same cut-off of 50 ng/mL for rivaroxaban, apixaban and dabigatran [15]. For this reason, the diagnostic test evaluation in our study was conducted using the three thresholds (< 30 ng/mL, < 50 ng/mL and <100 ng/mL).

Sensitivity and specificity of PT and aPTT were threshold-dependent. PT is indeed frequently described as an adequate test for rivaroxaban monitoring [4, 5, 24, 25]. Lim et al., in 2016, using the same reagent for PT assay, demonstrated a sensitivity and NPV above 90% for the cut-off of 50 ng/mL [15]. These results corroborate our findings. However, the observed differences in specificity and PPV can be attributed to the group studied. In addition to this influence, we believe that the differences in diagnostic accuracy related to aPTT may depend on the reagent used.

dRVVT screen and confirm tests presented maximum sensitivity and NPV, i.e., 100%, regardless of the threshold used. This would undoubtedly the safest test for exclusion of plasmatic concentration associated to hemorrhagic risk when the results are normal. The number of false positive results in relation to the total of exams for dRVVT screen and confirm varied from 32/80 to 51/80. These results are similar to the literature [7, 26].

Gosselin et al. [27], Exner et al. [11] described dRVVT as highly sensible for the presence of rivaroxaban. In fact, the authors described the application of dRVVT for many DOACs. Douxfils et al. [12] discussed also that dRVVT could be useful to assess pharmacodynamics of DOACs, with the advantage that it is a single test applied to different DOACs.

Gosselin et al. [27] describe that anticoagulants cause false results not only in coagulometric tests, as in the investigation of lupus anticoagulant, for example, but also in chromogenic tests. The interference of rivaroxaban on dRVVT was also described by other authors [26, 28, 29]. It is important to highlight that the sensitivity and specificity of a quantitative test are dependent on the cut-off value above or below which the test is positive [30].

Based on our results, we suggest that the patients with hemorrhagic risk (those with dRVVT screen and confirm tests above normality, that have sensitivity and low specificity) be submitted to a second test with higher diagnostic specificity (anti-Xa test calibrated with rivaroxaban or, in the absence of this, PT). This would allow the reduction of false positives and inadequate therapeutic interventions.

The potential limitations of this study must be considered, specially the reduced and highly homogeneous and clinically stable sample of patients, which might not be the case of other emergency settings. This must be considered in light of the fact that PPV and NPV are highly dependent on the disease prevalence.

## Conclusions

In conclusion, our findings showed that there is a correlation between the tests studied and the plasma concentration of rivaroxaban, confirming the existing description in the literature. In addition, as shown by the sensitivity and specificity results, our study suggests the applicability of the tests evaluated in the screening of plasmatic concentration associated to hemorrhagic condition in patients using rivaroxaban. However, it is crucial that the laboratory informs the attending physician about the diagnostic limitations of this group of tests in the evaluation of patients using anticoagulants, using an appropriate and clear note in the lab results report. Additionally, our results show a cut-off dependent behavior of the tests that must be better investigated in future studies.

## Abbreviations

ACS: Acute coronary syndrome
AF: Atrial fibrillation
anti-FXa: Anti factor X
aPTT: Activated partial thromboplastin time
C: Confirmatory
dRVVT: Dilute Russell viper venom time
PT: Prothrombin time
S: Screen
TT: Thrombin time
VTE: Venous thromboembolism
